# Improving Response Rates to EGFR-Targeted Therapies for Head and Neck Squamous Cell Carcinoma: Candidate Predictive Biomarkers and Combination Treatment with Src Inhibitors

**DOI:** 10.1155/2009/896407

**Published:** 2009-07-14

**Authors:** Ann Marie Egloff, Jennifer Rubin Grandis

**Affiliations:** ^1^Department of Otolaryngology, University of Pittsburgh, Pittsburgh, PA 15213, USA; ^2^Department of Pharmacology, University of Pittsburgh, Pittsburgh, PA 15213, USA; ^3^Eye and Ear Institute, Suite 500 200 Lothrop Street, Pittsburgh, PA 15213, USA

## Abstract

The epidermal growth factor receptor- (EGFR-) directed antibody, cetuximab, was FDA-approved for the treatment of squamous cell carcinoma of the head and neck (SCCHN) in 2006. Additional EGFR-targeting agents in clinical development for SCCHN include other EGFR-directed antibodies, tyrosine kinase inhibitors and antisense DNA. Although the majority of SCCHN overexpress EGFR, SCCHN clinical responses to EGFR-targeting agents have been modest. Molecular predictors for SCCHN response to EGFR-targeted therapies have not been identified. However, molecular correlate studies in lung cancer and colon cancer, which have EGFR-targeted therapeutics FDA-approved for treatment, may provide insights. We describe candidate predictive markers for SCCHN response to EGFR-targeted therapies and their prevalence in SCCHN. Clinical response will likely be improved by targeted therapy combination treatments. Src family kinases mediate EGFR-dependent and -independent tumor progression pathways in many cancers including SCCHN. Several Src-targeting agents are in clinical development for solid malignancies. Molecular correlate studies for Src-targeting therapies are few and biomarkers correlated with patient response are limited. Identifying SCCHN patients who will respond to combined EGFR- and Src-targeting will require further characterization of molecular correlates. We discuss rationale for EGFR and Src co-targeting for SCCHN treatment and describe recent clinical trials implementing combined Src- and EGFR-targeted therapeutics.

## 1. Introduction

Ninety-percent of head and neck cancers are squamous cell carcinomas (SCCHN) involving the mucosal surfaces of the oral cavity, pharynx, and larynx. The overall relative 5-year survival rates for cancers of the oral cavity/pharynx and larynx are estimated to be 58.3% and 64.5%, respectively [1]. Morbidities associated with SCCHN and its treatments are significant and include eating and swallowing difficulties. Targeted therapies for SCCHN are under active investigation with the goals of reducing SCCHN morbidity and mortality. 

Targeted therapeutics were conceptualized as a means of exploiting specific molecular alterations associated with cancers in order to selectively kill transformed cells and spare normal, healthy tissues. Targeted therapies are anticipated to have fewer associated toxicities than standard chemotherapies, which rely predominately on increased rates of cell division to enhance killing of the tumor cells compared to healthy tissues. For tumors that are treated with radiation and/or surgery, targeted therapies delivered systemically also have the potential to eliminate micrometastases that might not be eliminated with radiation therapy (RT) and/or surgery. In addition to reduced toxicity and treatment of undetected disease, it is hypothesized that effective targeted therapy may interfere specifically with processes that the cancer is dependent upon and be more effective than conventional therapies. 

The epidermal growth factor receptor (EGFR) was anticipated to be a good drug target for SCCHN treatment because the majority of SCCHN overexpress EGFR [2, 3], and higher tumor levels of EGFR are associated with poorer clinical outcomes [4, 5]. EGFR participates in SCCHN autocrine stimulation, and overexpression of EGFR and its primary ligand in humans, transforming growth factor alpha (TGF-*α*), have been correlated with poor outcomes for patients receiving therapy [5]. Cetuximab (Erbitux; ImClone Systems), a chimeric monoclonal IgG1 antibody directed against EGFR, was FDA-approved for the treatment of SCCHN in combination with RT for locally or regionally advanced disease and as a monotherapy for recurrent or metastatic SCCHN patients who have failed prior platinum-based therapy [6]. In addition to antibodies directed against EGFR, small molecule tyrosine kinase inhibitors (TKI) of EGFR and EGFR antisense agents are currently under active clinical investigation for SCCHN treatment. EGFR-targeted therapeutics delivered as monotherapies for treatment of SCCHN have demonstrated fewer toxicities compared to combined modality treatment regimens but only marginal clinical response (4–10%) [7, 8]. In general responses to EGFR-targeted therapies in SCCHN clinical trials have been modest.

Improving clinical response rates will involve (1) identifying SCCHN patients who are likely to respond to EGFR-targeted therapies, (2) developing effective combinations of targeted therapies, and (3) correctly identifying patients who will respond to specific targeted agents applied alone or in combination. Our understanding of the factors contributing to targeted therapy response now extends beyond the molecular alterations of the tumor to include host genetic variation. In this review, we will summarize molecular data correlated with clinical response to EGFR-targeted therapies and discuss factors that may be considered for identifying responsive SCCHN patients. Preclinical evidence suggests that Src family kinase-targeted agents administered in combination with EGFR-targeted therapies may demonstrate improved clinical response over EGFR-targeted agents alone. Here we also provide rationale for combining EGFR- and Src-targeted therapeutics for treatment of SCCHN, discuss published EGFR- and Src-combination treatment preclinical data, and summarize completed and ongoing clinical trials in solid tumors evaluating Src-targeted therapies in combination with EGFR-targeted therapies.

## 2. EGFR-Targeted Therapies for SCCHN

There are several EGFR-targeted therapies in clinical development for SCCHN, and these agents are described in Table 1. These inhibitors fall into two primary categories: EGFR-directed antibodies and EGFR tyrosine kinase inhibitors. EGFR-directed antibodies include cetuximab, nimotuzumab (YM Biosciences), panitumumab (Amgen), and zalutumumab (GenMab). EGFR-targeted tyrosine kinase inhibitors include erlotinib (Genetech and OSI Pharmaceuticals) and gefitinib (AstraZeneca). In addition to EGFR-targeted kinase inhibitors, inhibitors with broader target specificities are also in Phase II or III development for SCCHN including lapatinib (GlaxoSmithKline), which is a dual EGFR/HER2 inhibitor, and zactima (AstraZeneca), which targets VEGFR2 and RET in addition to EGFR (Table 1). More recently antisense therapy targeting EGFR has been evaluated in a Phase I clinical trial by our group [9]. 

Results of clinical trials for EGFR-targeted therapies cetuximab, nimotuzumab, gefitinib, and erlotinib used alone or in combination with conventional treatments for SCCHN have been reviewed by us and others and will not be described in detail here [10–12]. A Phase I study of panitumumab in combination with chemoradiotherapy involving 19-treatment-naïve patients with stage III/IV head and neck cancer reported an 87% complete response rate among the 15 evaluable patients and no grade 3 or 4 chronic toxicities [13]. A Phase I study of lapatinib in combination with chemoradiation in 31 patients with locally advanced SCCHN reported an overall response rate of 81% with radiation-associated mucositis, dermatitis, lymphopenia, and neutropenia as the most common grade 3 or 4 adverse events [14]. Our Phase I study of intratumoral delivery of EGFR antisense DNA in 17 patients with advanced, refractory SCCHN was associated with no grade 3 or 4 or dose-limiting toxicities and a clinical response rate (complete response and partial response by modified RECIST criteria) of 29% [9]. The current phase of clinical development for each of these agents is presented in Table 1.

## 3. Predictors of Response to EGFR-Targeted Therapies

To date, no molecular marker has been identified to correlate with SCCHN response to EGFR-targeting in patients. SCCHN tumor expression of the truncated form of EGFR, EGFR variant III (vIII), which lacks the ligand binding domain, occurs in up to 40% of SCCHN tumors and confers resistance to EGFR-targeted monoclonal antibodies in SCCHN preclinical models [15]. However, EGFR vIII expression and resistance to EGFR-targeted therapies in SCCHN patients has not been described. Molecular correlates of clinical response/nonresponse to EGFR-targeted therapies have been identified for colon and lung cancers. For example, the treatment of lung cancer with the EGFR tyrosine kinase inhibitor gefitinib demonstrated effective responses in a subset of patients whose lung cancers were subsequently found to harbor EGFR kinase activating mutations [16, 17]. Importantly, EGFR activating mutations do not appear to be frequent in SCCHN [18, 19]. Therefore, some of these molecular correlates, such as the EGFR tyrosine kinase activating mutations are not applicable to SCCHN because the frequency of these mutations is very low and will not be discussed further in this review. However, other molecular correlates of response to EGFR-targeting agents have been described for lung cancer and colorectal cancers including EGFR gene amplification, other somatic tumor mutations and patient genetic variations. These biomarkers have potential utility as predictive markers for SCCHN.

### 3.1. Tumor EGFR Gene Amplification

EGFR gene amplification occurs in SCCHN, and the rate of reported EGFR gene amplification in SCCHN varies substantially (Table 2). To date, there are no published reports evaluating EGFR gene amplification for association with SCCHN patient response to EGFR-targeted therapies. However, EGFR gene amplification has been reported to be positively associated with response to EGFR-directed antibody therapies in clinical trials for nonsmall lung cancers (NSCLC) and colorectal cancers. In a phase II study of 229 NSCLC patients with advanced-stage NSCLC treated with cetuximab plus chemotherapy, 76 patient tumors were evaluated for EGFR gene amplification by FISH and disease controls rate (complete response/partial response and stable disease) was found to be significantly higher in patients with FISH-positive tumors compared to FISH-negative tumors (81% versus 55%, *P* = .02). In this same study, median progression-free survival was also significantly longer for patients with FISH-positive tumors compared to FISH-negative tumors (6 months versus 3 months, *P* = .0008) [20]. Several studies have reported positive associations between EGFR gene amplification and metastatic colorectal cancer response to EGFR-directed antibodies [21–23]. 

EGFR gene amplification in SCCHN has been reported range between 10–58% of SCCHN (Table 2) [24–32]. The range of reported prevalence of EGFR gene amplification may be due to differences in expression by tumor anatomical site. However, several methods were used to assess EGFR gene amplification, including fluorescence in situ hybridization (FISH) and quantitative real-time polymerase-chain reaction- (Q-PCR-) based assays. In addition, different scoring methods were employed in the studies presented in Table 2, some of which included polysomy in the definition for EGFR amplification and others did not. These differences in methodologies likely contribute to the variation in reported rates of EGFR gene amplification in SCCHN. The presence of EGFR gene amplification in a substantial portion of SCCHN and the previously reported associations between EGFR gene amplification and response to EGFR-targeted therapies in other cancers suggest that EGFR gene amplification may be a predictive marker for response to EGFR-targeted therapies in SCCHN. When evaluating EGFR gene amplification for correlation with response to EGFR-targeted agents, it will be important to develop consensus definitions of EGFR gene amplification. 

EGFR gene amplification has not consistently been reported to correlate with EGFR protein levels although a plausible mechanism for gene amplification without protein overexpression is lacking [25, 28–30] (Table 2). In NSCLC and colon cancers a positive association between EGFR gene amplification and protein expression has also not been consistently observed [44, 45]. Importantly, EGFR gene amplification status, but not EGFR tumor protein levels, is associated with response to EGFR-targeted therapies in NSCLC and colorectal cancers. These discrepancies likely reflect the semiquantiatitve and variable methods of assessing gene amplification and protein expression levels in various laboratories. The characterization of EGFR gene amplification in SCCHN patients treated with EGFR-directed antibodies and the testing of association with response to therapy will be of interest.

### 3.2. Tumor KRAS/HRAS Mutations

Ras proteins are small GTPases that regulate signal transduction pathways leading to cell growth, differentiation, and survival. Three RAS genes produce four Ras proteins, KraS 4A, KraS 4B, H-Ras, and N-Ras, that are more than 90% homologous but demonstrate a high degree of tumor-type mutation specificity [46]. KRAS mutations have been reported by several independent groups to be negatively associated with response to EGFR tyrosine kinase inhibitors in lung cancer and EGFR-directed antibodies in colon cancer. A metaanalysis including 17 NSCLC EGFR tyrosine kinase inhibitor clinical studies with 1008 patient tumors and 8 metastatic colorectal cancer (mCRC) studies of anti-EGFR monoclonal antibody-based therapies in 817 mCRC patients found that KRAS mutations were significantly associated with absence of response to EGFR-targeted therapies for these cancers [47]. KRAS mutations are especially important predictors of unresponsiveness to EGFR-directed antibodies in colorectal cancers with EGFR gene amplification [48]. Reported rates of KRAS mutations in NSCLC range between 8–20% with higher rates reported for adenocarcinoma compared to squamous cell carcinoma histologies [49, 50]. KRAS mutations occur in approximately 30% of colon cancers [51]. 

KRAS mutations are relatively rare in SCCHN. Only one published report has described KRAS mutations in SCCHN to date, and in this analysis of 29 oral squamous cell carcinoma tumors, 9 (6.9%) were found to harbor KRAS mutations [24]. According to the Catalogue of Somatic Mutations in Cancer (COSMIC) database from the Sanger Institute, KRAS mutations occur in only approximately 3% of all cancers of the oral cavity, pharynx, or larynx while HRAS mutations occur in 10% of these cancers (Table 2) [34]. These rates are similar to data from the Genetic Alterations in Cancer (GAC) database presented in Table 2 [33]. The recent finding that a mouse knock-in model expressing HRAS from the KRAS chromosomal context accumulated HRAS mutations and resulted in increased lung tumorigenicity suggests that tissue-specific expression of KRAS and HRAS likely contributes to tumor-type specificity of mutations in these two genes [52]. To date, only one published study of HRAS mutations SCCHN reported a 22% rate of mutations in oral squamous cell carcinoma (Table 2) [35]. Mutations in HRAS are likely to be more common in SCCHN than KRAS mutations and may be important correlates for lack of response to EGFR-targeted therapies in SCCHN.

### 3.3. Tumor PI3K-AKT Pathway Mutations

Phosphatidylinositol 3-kinases (PI3Ks) are heterodimeric kinases composed of regulatory and catalytic subunits that are involved in the control of cell proliferation, survival, and motility. The PI3K catalytic subunit, P110alpha (PIK3CA) has been reported to be somatically mutated and activated in several cancers including SCCHN. Activation of PIK3CA leads to plasma membrane recruitment and activation of Akt and downstream survival mechanisms. PIK3CA mutations have been reported to be associated with resistance to EGFR-targeted monoclonal antibodies in patients with metastatic colorectal cancers (mCRC). In a study involving 110 patients with mCRC, PIK3CA mutations were found to be significantly associated with reduced objective response rates following treatment with cetuximab or panitumumab (*P* = .038) and shorter progression-free survival (*P* = .035) [53]. PIK3CA mutations have been reported to occur in up to 8% of SCCHN as summarized in Table 2 [36–39].

PI3K signaling is inhibited by the activity of the phosphatidylinositol phosphatase, PTEN. PTEN acts as a tumor suppressor by negatively regulating the Akt signaling pathway. PTEN mutations occur in colorectal, lung, and head and neck cancers. Additionally, loss of PTEN expression occurs by mechanisms including promoter methylation and silencing or loss of heterozygosity. In SCCHN, PTEN mutations are not common (Table 2) [40–43], and loss of heterozygocity of PTEN has been reported to occur in approximately 12% of SCCHN [42]. Though the association with response to EGFR-targeted therapy in mCRC and loss of PTEN expression does not appear to be as strongly correlated as response and PIK3CA mutations [53], the consideration of both tumor PTEN expression status and PIK3CA mutation status may contribute to predicting response to EGFR-targeted therapies in SCCHN.

### 3.4. EGFR Polymorphisms

Several EGFR polymorphisms have been reported to be associated with differential response to EGFR-targeted therapies. In lung cancer, shorter EGFR intron 1 CA repeat polymorphism has been reported to be associated with improved response to gefitinib in two independent studies [54, 55]. In one study involving 70 patients with advanced NSCLC, patients with fewer than 17 CA repeats at either allele had significantly longer survival following treatment with gefitinib than patients having both alleles greater than 16 CA repeats (*P* = .039) [54]. Fewer EGFR intron 1 CA repeats were also significantly associated with mCRC patient response to cetuximab-based treatment in a study involving 110 mCRC patients receiving combined cetuximab-irinotecan salvage therapy [56]. An independent study of 139 NSCLC patients with WHO performance status of 0 or 1 who received gefitinib reported that patients with the EGFR haplotype of −216G/−191C had significantly worse survival with a hazard ratio of 1.85 (95% CI: 1.09 to 3.12) after adjusting for performance status, previous platinum treatment, skin rash, and diarrhea [57]. The EGFR intron 1 CA repeat polymorphism has been reported to affect EGFR basal transcription with higher transcription rates reported in individuals with fewer CA repeats [58, 59]. Differential promoter activity has also been reported for the two most common EGFR haplotypes at the −216G > T and −191C > A with the −216G/−191C haplotype having lower promoter activity and mRNA expression [60, 61]. These studies, therefore, indicate that patient EGFR polymorphisms associated with higher EGFR expression are more likely to respond to EGFR-targeted therapies. 

The presence of the EGFR K521R variant has also been found to be associated with significantly improved progression-free survival (PFS) and overall survival (OS) in 32 EGFR-positive mCRC patients treated with cetuximab in combination with irinotecan [62]. Patients with the K521R variant had significantly longer PFS than patients with wild-type EGFR, 5.7 months versus 3.2 months, respectively, (*P* = .04, log rank test) and OS, 20.1 months versus 13.8 months, respectively, (*P* = .03) [62]. This EGFR variant, which resides in the extracellular domain of EGFR, has reduced ligand-binding, growth-stimulation, and kinase activity in vitro for the 521K variant. These findings suggest that EGFR polymorphisms have the potential to be correlated with response to EGFR-targeted therapies in SCCHN.

### 3.5. FC*γ*RIIa and FC*γ*RIIIa Polymorphisms

Cetuximab, a chimeric monoclonal IgG1 anti-EGFR antibody (Table 1), may exert its antitumor effects via several mechanisms including antibody-dependent cell mediated cytotoxicity (ADCC). The fragment c (Fc) portion of IgG1 antibodies can be recognized by the Fc gamma receptors (FC*γ*R) on immune effector cells to induce ADCC. Polymorphisms in FC*γ*RIIIa have been shown to be associated with differential response to cetuximab in mCRC patients in clinical studies and to SCCHN cell lines in vitro [63–65]. The FC*γ*RIIIa polymorphism-V158F variant 158V was found to have higher cetuximab-mediated ADCC in vitro [64, 65]. The 158V variant was also associated with longer PFS in a study involving 69 mCRC patients treated with cetuximab plus irinotecan [63]. These findings indicate that FC*γ*RIII variants may contribute to response to cetuximab in SCCHN patients.

The ability to correctly predict which patients will respond to which EGFR-targeted therapy will improve clinical response and reduce treatment-associated toxicities for these patients. However, the minority of SCCHN patients have responded to EGFR-targeted therapies in clinical trials, indicating that even if patients likely to respond to EGFR-targeted therapy were identified, they would represent a small portion of SCCHN patients. Even though the majority of SCCHN cancers overexpress EGFR, these tumors are not solely dependent upon EGFR activity. This is likely due to the presence of preexisting or treatment-induced compensatory signaling pathways. Because EGFR is activated in SCCHN and response to EGFR-targeted therapies has been demonstrated in clinical trials, it is reasonable to consider targeted therapies to be used in combination with EGFR-targeted therapeutics. Molecular signaling pathways in SCCHN that can be activated independently of EGFR include pathways initiated by G-protein-coupled receptors, integrins, and other receptor tyrosine kinases. Many of these pathways share Src family kinases (SFK) as downstream mediators of signaling. For these reasons, SFK have been identified as viable candidates for targeting in combination with EGFR. The combination of SFK- and EGFR-targeted agents for treatment of SCCHN is anticipated to have improved clinical efficacy compared to EGFR-targeting agents alone.

## 4. Src Family Kinases in SCCHN

Eight Src nonreceptor protein tyrosine kinase family members are expressed in humans: c-Src, Blk, Fgr, Fyn, Hck, Lck, Lyn, and c-Yes. c-Src, Fyn, Lyn, and c-Yes are broadly expressed, while Blk, Fgr, Hck, and Lck expression is primarily restricted to hematopoietic cells [66]. Src kinases have been implicated in normal cellular functions including cell adhesion, migration, angiogenesis, survival, proliferation, and differentiation [67, 68]. When these processes are inappropriately regulated, they can contribute to tumorigenesis, tumor progression and metastases. In SCCHN models, Src kinases are activated in response to EGFR stimulation [69], associate with EGFR [69], and have reduced activity following EGFR inhibition in vitro [70]. Src kinases are also upstream activators of EGFR and other receptor tyrosine kinases (RTKs). Following G-protein-coupled receptor (GPCR) stimulation, Src kinases are activated, resulting in the release of RTK ligands [71, 72]. In addition to RTKs and GPCRs, Src kinases are also activated by integrins in SCCHN [73]. Src kinases, therefore, are involved in the autocrine/paracrine stimulation of SCCHN and mediate EGFR-dependent and EGFR-independent signaling events.

Of the Src family kinases (SFK), c-Src is the most studied and most often implicated in cancer. Elevated c-Src protein and/or kinase activity has been reported for cancers of the lung, colon, breast, ovary, and pancreas in addition to head and neck cancers [68, 74]. c-Src is rarely mutated in cancer [74–76]. Therefore, increased activity of upstream signaling components and/or decreased activity of c-Src negative regulators are likely causes of c-Src activation observed in many epithelial cancers. 

The expression and activation of specific SFK in SCCHN are less well understood. The SFK c-Src, Fyn, Lyn, and c-Yes are activated in SCCHN cell lines in vitro following stimulation with the EGFR ligand TGF-*α* [69], and these SFK likely play roles in SCCHN. At least one group has reported differential response of SFK to integrin *β*_6_ signaling following simulation with fibronectin, the integrin *β*_6_ ligand, in oral squamous cell carcinoma cell lines. Integrin *β*_6_, which is neoexpressed in SCCHN, has been found to activate Fyn but not c-Src or c-Yes in SCCHN upon ligation with fibronectin, leading to Fyn-dependent activation of the Raf-ERK/MAPK pathway [73]. The murine knock-out models of specific SFK provide insights into the different roles of the individual SFK. The functions of some of the SFKs are redundant, at least regarding mouse development, as evidenced by lack of phenotype for single knock-out models of c-yes, hck, c-fgr, and blk [66]. The single knock-out murine models of lyn and lck had immune impairments, fyn knock-out mice exhibited defective brain development and impaired memory and immune functions, and c-src null mice developed a bone remodeling disease with excess accumulation of bone [66]. Therefore, some functions are likely shared between the four SFKs with a subset of functions that may be unique to selective SFK.

## 5. Src Family Kinases in SCCHN Invasion and Progression

Mortality from SCCHN is usually associated with tumor invasion and locoregional metastases. The major site of SCCHN metastases is locoregional lymph nodes, and presence of neck lymph node metastases is universally accepted as the most important prognostic indicator for SCCHN. The development of metastases requires that cells move from the primary tumor and invade surrounding tissues. Invasion by tumor cells is preceded by the loss of cell adhesion and the gain of mesenchymal features in a process similar to events that occur in development termed epithelial-mesenchymal transition (EMT) [77]. EMT is accompanied by the loss of E-cadherin, which is a principal component of cell adhesion complexes, and the gain of mesenchymal characteristics including expression of vimentin [77]. 

The activation of Src kinases has been shown to be involved in EMT in cancer [78]. More recently, a study evaluating 50 primary SCCHN tumors for activated phospho-Src (P-Src), E-cadherin, and vimentin expression by immunohistochemical staining found increased P-Src, decreased E-cadherin, and presence of vimentin expression in SCCHN tumors to be significantly associated (*P* < .05) with morphologies associated with aggressive cancers including penetrating invasive fronts, poor or sarcomatoid differentiation, and lymph node metastases [79]. It is important to note that the P-Src antibody used in this study recognizes several activated SFKs and is not specific for P-c-Src. In addition to studies in SCCHN tumors, preclinical studies indicate that SFKs are involved in SCCHN migration and invasion. Our group found that blockage using an Src-specific inhibitor A-419259 resulted in decreased invasion and growth of SCCHN cell lines in vitro following stimulation with a GPCR ligand, gastrin-releasing peptide [72]. An independent group reported that dasatinib (Sprycel, BMS-34825; Bristol-Meyers Squibb), a dual Src/Abl kinase inhibitor (Table 3), inhibited migration and invasion in vitro in all 8 SCCHN cell lines evaluated [80]. Together these studies implicate an important role or roles for SFK in tumor migration and invasion, which are associated with increased mortality in SCCHN. Which SFKs are activated in SCCHN migration and invasion is currently not known. Importantly, epithelial tumor cells including SCCHN that have undergone EMT and acquired mesenchymal characteristics are more resistant to EGFR-targeted therapies than tumor cells that have epithelial characteristics [81]. Combining Src-targeted agents with EGFR-targeted therapies may be more effective than EGFR-targeted therapies alone for the control of SCCHN locoregional metastases.

## 6. Src-Targeting Agents in Clinical Development

Several small molecule inhibitors of c-Src and SFK are currently in clinical development for solid tumors including dasatinib (Sprycel, BMS), AZD0530 (AstraZeneca), bosutinib (SKI-606; Wyeth), XL999 (Exelixix), and KX01 (Kinex) (Table 3). All of these inhibitors are reversible inhibitors, and all except KX01 are ATP-competitive inhibitors (Table 3). These inhibitors differ primarily in their target specificities. Dasatinib and XL999 target several known kinases in addition to SFK (Table 3), while AZD0530 and bosutinib are dual SFK/Abl inhibitors. Dasatinib, which was FDA-approved for treatment of nonsolid tumors in June 2008, and AZD0530 are in Phase II clinical trials for SCCHN. Bosutinib and XL999 are in Phase II clinical trials for other cancers (Table 3). However, XL999, which inhibits VEGFR, PDGFR and FGFR in addition to Src kinases, was associated with serious cardiovascular toxicities in Phase I and II clinical trials [82–84]. Exelixis suspended new patient enrollment in the ongoing XL999 clinical trials in November 2006. A new addition to Src inhibitors in clinical development includes the c-Src substrate competitive inhibitor, KX01, which is currently being tested in phase I clinical trials. KX01 is exquisitely specific for c-Src whereas other Src-targeting agents inhibit other SFK in addition to c-Src [85–87]. To date there are no reports of Src-targeted therapeutics in SCCHN clinical trials or molecular predictors of response to Src-targeted therapies in patients with solid malignancies.

## 7. Cotargeting of EGFR and Src Family Kinases in Patients

Combining EGFR- and Src-targeted therapies for SCCHN is supported by results from preclinical studies. Our group reported that combined AZD0530 and gefitinib treatment of SCCHN cell lines in vitro resulted in significantly reduced cell growth and invasion compared to single agent treatments [88]. De novo and acquired resistance to cetuximab are means by which SCCHN patients fail therapy.

SCCHN and NSCLC preclinical models selected for resistance to cetuximab in vitro have been reported to have high levels of activated SFK and to have decreased PI3K/Akt activity following dasatinib treatment [89]. Interestingly, these cetuximab resistant cells were found to be resensitized to cetuximab following treatment with dasatinib [89]. These data in addition to our current understanding that many EGFR-independent cell signaling pathways, including GPCR- and integrin-initiated pathways, are modulated at least in part by SFK provide the rationale for the combined targeting of EGFR and SFK for treatment of SCCHN. 

To date, there are no published reports of combined EGFR- and Src-targeted therapies for treatment of patients with solid tumors. Three clinical trials combining EGFR- and Src-targeted therapies for upper aerodigestive cancers are currently ongoing. A Phase I trial combining dasatinib with erlotinib in patients with recurrent NSCLC is ongoing at the H. Lee Moffitt Cancer Center and Research Institute (NCT00444015, ClincalTrials.gov). A Phase I/II study in NSCLC also combining dasatinib with erlotinib is ongoing at M.D. Anderson Cancer Center (NCT00826449, ClinicalTrials.gov). Our group will soon open a Phase 0 biomarker modulation study combining erlotinib with dasatinib for patients with SCCHN or NSCLC (NCT00779389, ClinicalTrials.gov). Results from these trials are not yet available. Our group recently completed a Phase I trial combining cetuximab with dasatinib for treatment of advanced solid malignancies (NCT00388427, ClinicalTrials.gov). Seventeen of 25 patients enrolled in our Phase I study were evaluable for response, and 9 had stable disease while head ache was a primary toxicity [90]. We have evaluated molecular correlates in these patients and found that P-SFK levels in peripheral blood mononuclear cells were transiently reduced following daily dasatinib dosing (unpublished data). In addition, we found that EGFR, TGF-*α* and amphiregulin plasma levels were altered following treatment (unpublished data). A Phase II study combining dasatinib and cetuximab for treating SCCHN patients is planned at the University of Pittsburgh Cancer Institute. Results from molecular correlate studies from this trial and others will be of great importance as the SCCHN medical and research communities work to identify predictive molecular markers of response to these therapies.

## 8. Summary and Future Directions

Despite the ubiquitous expression of EGFR in SCCHN, clinical responses to EGFR targeting agents, particularly, when administered as single agents, has been modest. Cetuximab was FDA-approved in 2006 for the treatment of newly diagnosed SCCHN in combination with radiation and recently extended to the recurrent/metastatic population in combination with chemoradiotherapy. However, in most of these trials, expression levels of EGFR in the tumor have not correlated with response to cetuximab and no single biomarker to date in baseline tissue has been proven to predict response to EGFR targeting agents. Comprehensive genomic and proteomic studies of baseline tissue are required in the context of clinical trials to begin to identify potential markers of clinical activity. Since EGFR signaling involves intracellular interactions with other oncogenic pathways in SCCHN preclinical models, it is plausible that cotargeting of EGFR in conjunction with blockade of these pathways may be beneficial. Src family kinases represent a potential pathway for targeting, especially given the FDA-approval of the Src kinase inhibitor dasatinib for hematopoietic malignancies. Studies are underway to test this hypothesis in SCCHN patients. Challenges include: (1) the difficulty of testing antiinvasive/antimetastatic agents in clinical trial settings, and (2) the possibility that RECIST criteria may not reflect decreased tumor proliferation, metabolism, or increased apoptosis as evidence by studies that have incorporated PET tracers. More relevant endpoints in EGFR-/Src-targeted trials than tumor shrinkage may include time to progression or overall survival. This may be especially relevant for locoregional recurrence/metastases in SCCHN [91].

## Figures and Tables

**Table 1:** EGFR-targeted therapies in clinical development for SCCHN.

Agent		Sponsor	Class	FDA-approval	Clinical trial phase for SCCHN
Antibodies					

Cetuximab	C225, Erbitux	ImClone Systems	Chimeric IgG1	SCCHN; colorectal cancers	III
Nimotuzumab	h-R3	YM Biosciences	Humanized IgG1	—	IV Advanced disease; II Locally advanced disease
Panitumumab	ABX-EGF; Vectibix	Amgen	Fully human IgG2	Colorectal cancers	III
Zalutumumab	HuMax-EGFR	GenMab	Fully human IgG1	—	III

Tyrosine kinase inhibitors					

Erlotinib	Tarceva; OSI-774	Genetech and OSI Pharmaceuticals	Reversible ATP competive	Lung cancer	III
Gefitinib	ZD-1839; Iressa	AstraZeneca	Reversible ATP competive	Lung cancer, relabeling limits	III
Lapatinib	Tykerb	GlaxoSmithKline	Reversible ATP competitive dual EGFR/Her2	Breast cancer	III
Zactima	ZD6474	AstraZeneca	Reversible ATP competitive VEGFR-2, EGFR and RET	—	II

**Table 2:** Candidate predictive markers for SCCHN response to EGFR-targeted therapies.

Tumor molecular marker	Study/ reference	Tumor type(s)	N tumors assessed	N tumors with molecular marker	Assay method	Positive scoring definition(s)	Associated with EGFR tumor levels
EGFR gene amplification	Sheu et al., 2009 [24]	OSCC	128	22 (17.2%)	FISH	>2.5 EGFR signals relative to Cen7 signal	Yes
	Ch’ng et al., 2008 [25]	SCCHN	39	18 (46%)	FISH	>2 EGFR signals relative to Cen7 signals or ≥15 EGFR copies per cell in ≥10% of cells	No
	Chiang et al., 2008 [26]	OSCC	42	14 (33%)	Q-PCR	≥2 EGFR gene copies relative to LINE1 element	No
	Temam et al., 2007 [27]	SCCHN	134	22 (17%)	Q-PCR (*n* = 134) and FISH (*n* = 16)	Q-PCR: >mean + 1.96 standard deviations of normal WBC EGFR gene copy number normalized to *β*-globin; FISH: ≥4 gene copies in 40% of cells or gene/chromosome ratio >2 or ≥15 gene copies in ≥10% of cells	No significant correlation between EGFR gene amplification by FISH and EGFR IHC expression
	Chung et al., 2006 [28]	SCCHN	75	43 (58%)	FISH	≥4 gene copies in 40% of cells or gene/chromosome ratio >2 or ≥15 gene copies in ≥10% of cells	No
	Hanawa et al., 2006 [29]	ESCC	106	53 (50%)	FISH	EGFR signal > Cen7 signal	Yes
	Mrhalova et al., 2005 [30]	SCCHN	33	7 (21%)	FISH		No
	Koynova et al., 2005 [31]	Larynx cancers	1080	112 (10.4%)	FISH	≥4 EGFR signals relative to Cen7 in ≥10% of cells	NA
	Freier et al., 2005 [32]	SCCHN	609	12.70%	FISH	≥8 EGFR signals relative to Cen7 in ≥10% of cells	NA

KRAS mutations	Sheu et al., 2009 [24]	OSCC	29	2 (6.9%)	Sequencing	KRAS Q61H mutation	NA
	Lea et al., 2007 [33]	ORAL cancers	122	5 (4%)	GAC database analysis	Somatic missense, nonsense, silent point mutations, frameshift and in-frame deletions and insertions	NA
	Forbes et al., 2008 [34]	Oral, pharynx, larynx cancers	937	24 (3%)	COSMIC database	Datamining of published reports and somatic mutation screening from Cancer Genome Project	NA

HRAS mutations	Forbes et al., 2008 [34]	Oral, pharynx, larynx cancers	686	75 (10%)	COSMIC database	Datamining of published reports and somatic mutation screening from Cancer Genome Project	NA
	Lea et al., 2007 [33]	ORAL cancers	170	19 (11%)	GAC database analysis	Somatic missense, nonsense, silent point mutations, frameshift and in-frame deletions and insertions	NA
	Anderson et al., 1994 [35]	ORAL cancers	35	6 (22%)	PCR and restriction length polymorphism analysis	Presence of appropriately altered restriction enzyme digested DNA fragment	NA

PI3KCA mutations	Murugan et al., 2008 [36]	SCCHN	37	2 (5%)	PCR and direct sequencing exons 9 and 20	Somatic missense, nonsense, frameshift, in-frame deletions, and insertions	NA
	Fenic et al., 2007 [37]	SCCHN	33	0 (0%)	PCR and direct sequencing exons 9 and 20	Somatic missense mutations	NA
	Qiu et al., 2006 [38]	SCCHN	38	4 (11%)	PCR and direct sequencing exons 1, 4, 5, 6, 7, 9, and 20	Somatic missense mutations	NA
	Kozaki et al., 2006 [39]	OSCC	108	8 (7%)	PCR and direct sequencing exons 9 and 20	Somatic missense mutations	NA

PTEN mutations	Shin et al., 2002 [40]	OSCC	86	4 (5%)	PCR and exon direct sequencing	Somatic missense, nonsense, silent point mutations, frameshift, in-frame deletions, and insertions	NA
	Poetsch et al., 2002 [41]	SCCHN	52	7 (13%)	PCR and exon direct sequencing	Somatic missense, nonsense, frameshift, in-frame deletions, and insertions	NA
	Mavros et al., 2002 [42]	OSCC	50	0 (0%)	PCR and exon direct sequencing	Somatic missense, nonsense, frameshift, in-frame deletions, and insertions	NA
	Shao et al., 1998 [43]	SCCHN	19	3 (16%)	PCR and exon direct sequencing	Somatic missense, nonsense, frameshift, in-frame deletions, and insertions	NA

Squamous cell carcinoma of the head and neck (SCCHN), oral squamous cell carcinoma (OSCC), esophageal squamous cell carcinoma (ESCC), fluorescence in situ hybridization (FISH), quantitative real-time polymerase chain reaction (PCR), centromere 7 (Cen7), Genetic Alterations in Cancer (GAC) database, and the Catalogue of Somatic Mutations in Cancer (COSMIC) database.

**Table 3:** Src-targeting agents in clinical development.

Agent		Sponsor	Target(s)	SFKs targeted (IC50)	Target site	Irreversible	Solid cancers in phase II or III clinical study∗	FDA approval (Date)	SCCHN clinical trial phase
Dasatinib	BMS-354825	Bristol-Myers Squibb	Src; Abl; c-Kit; PDGFR; others		ATP-binding	No	SCLC, NSCLC, breast, colorectal, head and neck, liver, melanoma, ovarian, pancreatic, sarcoma	Chronic myeloid leukemia (June 2006)	II

AZD0530		AstraZeneca	Src; Abl		ATP-binding	No	SCLC, NSCLC, breast, colorectal, head and neck, melanoma, osteosarcoma, ovarian, pancreatic, prostate	—	II

Bosutinib	SKI-606	Wyeth	Src; Abl		ATP-binding	No	Breast	—	—

KX01	KX2-391	Kinex	Src		Peptide-binding	No	(phase I)	—	—

XL999		Exelixis	Src, VEGFR, PDGFR, FGFR, FLT-3, others		ATP-binding	No	NSCLC, colorectal, kidney, ovarian	—	—

∗ClinicalTrials.gov solid tumors.
